# Diagnostic Yield of *Strongyloides stercoralis* Screening in Immunocompromised Patients and Those with Anticipated Immunocompromise in a Dutch Tertiary Hospital: A Retrospective Cohort Study

**DOI:** 10.4269/ajtmh.24-0803

**Published:** 2025-07-22

**Authors:** Elsemieke te Linde, Sonja E. van Roeden, Frans M. Verduyn Lunel, Leo G. Visser, Anke H. W. Bruns

**Affiliations:** ^1^ Department of Infectious Diseases, University Medical Center Utrecht, Utrecht, The Netherlands;; ^2^ Department of Internal Medicine, Medical Center Leeuwarden, Leeuwarden, The Netherlands;; ^3^ Department of Medical Microbiology, University Medical Center Utrecht, Utrecht, The Netherlands;; ^4^ LUCID, Subdepartment of Infectious Diseases, Leiden University Medical Center, Leiden, The Netherlands

## Abstract

Strongyloidiasis is a parasitic infection caused by the intestinal helminth *Strongyloides stercoralis* (*S. stercoralis*). It is prevalent in tropical and subtropical regions but increasingly diagnosed in nonendemic countries because of migration and travel. In immunocompromised patients, infection with *S. stercoralis* can lead to a potentially lethal hyperinfection syndrome. Consequently, several guidelines recommend screening immunocompromised patients for *S. stercoralis* IgG. We aimed to assess the yield of this screening in our tertiary hospital in the Netherlands, a nonendemic country. We performed a retrospective cohort study, in which we identified all immunocompromised patients and those expected to be immunocompromised who were tested for *S. stercoralis* IgG between January 2010 and April 2022. For these patients, we collected demographic and clinically relevant data from electronic patient files. *Strongyloides stercoralis* IgG test results were positive in 17/379 patients (4.5%), all of whom were born in an endemic country. Eosinophilia was present in 7/17 patients (41.2%). Over the years, the number of diagnostic tests performed for *S. stercoralis* IgG has increased significantly, but the number of positive test results per year has not. Focusing screening on patients who have lived in endemic regions and less on travelers will improve diagnostic yield. We recommend screening all immunocompromised patients from high-endemic countries and highly immunocompromised patients with relevant travel history. For less severely immunocompromised patients, screening should be individualized based on travel characteristics.

## INTRODUCTION

Strongyloidiasis is a chronic parasitic infection of humans caused by the intestinal helminth *Strongyloides stercoralis* (*S. stercoralis*). This roundworm infects people through direct contact with soil that contains the larvae. An estimated 30–100 million people worldwide are infected, and the infection is endemic in tropical and subtropical regions, as well as in countries with temperate climates.^1^ Because of migration and international travel, a growing number of people are diagnosed with strongyloidiasis in nonendemic countries.^2^^,^^3^

*Strongyloides stercoralis* has some unique features when compared with other soil-transmitted helminths. First, it can complete its lifecycle, also known as the “autoinfection cycle,” within the human host.^4^ This enables the helminth to persist in the host for decades, even in the absence of reinfection, thus sustaining a chronic infection. People with chronic strongyloidiasis are often asymptomatic or present with very nonspecific symptoms involving the skin, gastrointestinal tract, or respiratory tract.^5^ Second, in the case of reduced host immunity (e.g., due to immunosuppressant medication or organ transplantation), disruption in T helper type 2 cell-mediated immunity may stimulate the autoinfection cycle to dramatically increase the burden of parasites. This can lead to severe manifestations of the infection, such as *S. stercoralis* hyperinfection syndrome, in which the parasite undergoes uncontrolled proliferation and disseminates to virtually any organs other than the skin, lungs, and gastrointestinal tract.^6^^,^^7^ Although uncomplicated cases of *S. stercoralis* infection are easily treatable with oral ivermectine, hyperinfection syndrome is difficult to treat and has a fatality rate of more than 60% despite treatment.^8^^–^^10^ With an increasing number of immunocompromised patients in recent decades, more patients are at risk of this severe complication.^11^

Therefore, several guidelines recommend screening for chronic strongyloidiasis by assessing the presence of *S. stercoralis* IgG before starting with immunosuppressive therapy or upon the diagnosis of an immune-compromising disorder.^12^ However, the guidelines are ambiguous regarding indications for screening.^13^^–^^16^ Moreover, the efficacy of screening is unknown and depends on the population selection, which requires an adjusted approach depending on the specific geographic setting and background of the patients involved.

We set out to assess the yield of screening for strongyloidiasis in our tertiary hospital facility in the Netherlands over the past 12 years in a cohort of patients who are or will be immunocompromised in the near future. Apart from the seroprevalence of this cohort, we aimed to identify factors associated with a positive screening test result to optimize the current screening strategy in our facility.

## MATERIALS AND METHODS

### Study site and population.

We performed a retrospective, observational cohort study to assess the diagnostic yield of screening for *S. stercoralis* IgG in clinical practice at the University Medical Center Utrecht, a tertiary hospital in the center of the Netherlands. We selected patients who were serologically tested for *S. stercoralis* IgG between January 2010 and April 2022. Subsequently, we selected the patients who were or would become immunocompromised. In our institution, patients living with HIV who are from endemic countries and those who have traveled to these countries are routinely screened for *S. stercoralis*. For other patients, testing for *S. stercoralis* is not performed routinely. However, screening is advised for immunocompromised patients or transplant recipients who have traveled to or lived in endemic countries.

### Goals.

Our primary objective was to assess the proportion of *S. stercoralis* IgG in screened patients who are or will be immunocompromised in the near future, as well as to identify factors associated with a positive screening test result. The secondary objective was to describe the seroprevalence in our cohort over time.

### Data selection.

All sera screened for *S. stercoralis* IgG between January 2010 and April 2022 were automatically extracted from the laboratory information system and assessed. Patients who were not immunosuppressed or were not scheduled to start immunosuppressive therapy were excluded (*n* = 248). Other reasons for exclusion were as follows: age <18 years (*n* = 29) and active objection against usage of their data for research purposes (*n* = 18).

For the remaining patients, we collected data on age, sex, the date of the sample draw, repeated samples, country of birth, travel history, medical history, reason for (starting) immunosuppression, immunosuppressive medication, indication for screening, presence of eosinophilia, and clinically relevant symptoms from electronic patient files. All data were processed and stored anonymously in Castor EDC software (Castor EDC, Claymont, DE).

### Definitions.

An immunocompromised state was defined as the presence of one of the following conditions: any previous solid organ transplantation, recent (<2 years before inclusion) hematological stem cell transplantation, active hematological malignancy, primary immunodeficiency disorder, HIV infection, or the chronic use of systematic immunosuppressive drugs. Patients taking prednisone were only considered immunocompromised if they used more than 10 mg/day or a cumulative dose of >700 mg.^17^ For azathioprine, the minimum dose was 3.0 mg/kg/day, and for 6-mercaptopurine, the minimum dose was 1.5 mg/kg/day.^18^ Patients with a solid malignancy were considered immunocompromised when the last chemotherapy or the last immune therapy was within 4 weeks before the assessment of *S. stercoralis* IgG.

Patients who were screened because of the (intended) start of immunosuppressive medication or the diagnosis of an immunosuppressive condition were grouped (hereinafter referred to as (start)immunosuppression). However, patients with HIV were analyzed separately because their screening was protocol-driven at the time of HIV diagnosis or upon entering HIV care if there was anamnestic evidence of possible *S. stercoralis* exposure (Table 1).

**Table 1:** Characteristics of (anticipated) immunocompromised patients by outcome of *S. stercoralis* IgG screening

Characteristics	Total	*S. stercoralis*, IgG-Positive	*S. stercoralis*, IgG-Negative
Total, *n* (%)	379 (100)	17 (4.5)	362 (95.5)
Male sex, *n* (%)	222 (58.6)	9 (52.9)	213 (58.8)
Median age, years (IQR)	46 (32.0–58.0)	31 (28.5–44.0)	46 (33.0–58.0)
Screened before start immunosuppression	60 (15.8)	0 (0)	60 (16.6)
Immunocompromised	319 (84.2)	17 (100)	302 (83.4)
Reason for immunosuppression			
Medication	61 (19.1)	0 (0)	61 (16.9)
HIV	150 (47.0)	13 (76.5)	137 (37.8)
Hematological malignancy	71 (22.3)	2 (11.8)	69 (19.1)
Solid organ transplantation	21 (6.6)	1 (5.9)	20 (5.5)
HSCT	7 (2.2)	0 (0)	7 (1.9)
Primary immunodeficiency	5 (1.6)	0 (0)	5 (1.4)
Solid malignancy	2 (0.6)	0 (0)	2 (0.6)
Other	3 (0.9)	1 (5.9)	2 (0.6)
Reasons for screening*			
Origin from an endemic country	217 (57.3)	17 (100)	200 (55.2)
Travel history to ≥1 endemic country	219 (57.8)	6 (35.3)	213 (58.8)
Eosinophilia	46 (12.1)	6 (35.3)	40 (11.0)
HIV	113 (29.8)	7 (41.2)	106 (29.3)
(Start) immunosuppression	170 (44.9)	3 (17.6)	167 (46.1)
Symptoms	65 (17.2)	4 (23.5)	61 (16.9)
Other	6 (1.6)	0 (0)	6 (1.7)

Data presented as *n* (%) unless otherwise indicated. HSCT = hematopoietic stem cell transplantation; IQR = interquartile range; *S. stercoralis* = *Strongyloides stercoralis*.

* >1 reason per patient is possible.

**Figure 1.**
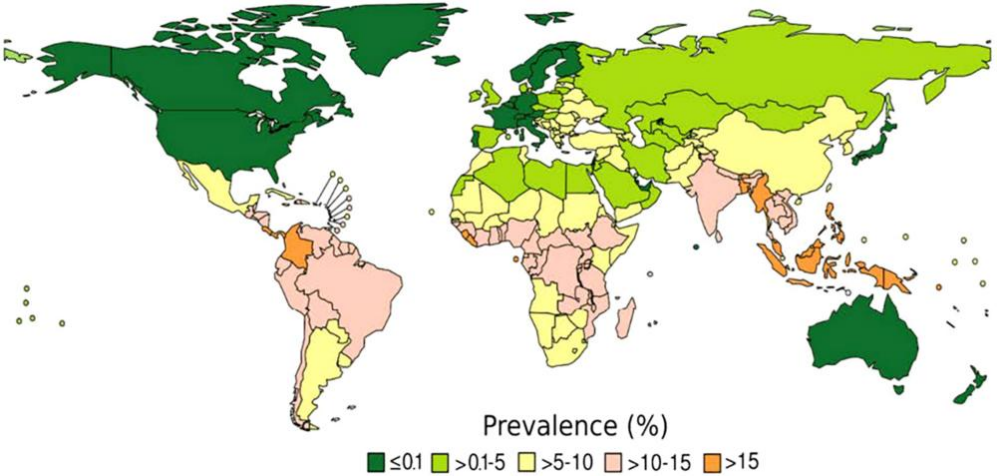
Prevalence of *Strongyloides stercoralis* infection worldwide. Figure taken from *A practical approach to screening for* Strongyloides stercoralis, Tropical Medicine and Infectious Disease, 2021. This figure was modified from: *The global prevalence of* Strongyloides stercoralis *infection*, Pathogens, 2020. All authors permitted the reproduction.

Originating from an endemic country was defined as being born or having lived >12 months in a country with a prevalence of *S. stercoralis* IgG of ≥5% among the general population (Figure 1).^19^ The same prevalence criteria were used to assess the history of traveling to an endemic country. The length of stay was not included because there is no set cutoff for the risk of acquiring strongyloidiasis defined in the literature. Therefore, any travel duration to endemic areas was included and considered a potential exposure. The presence of eosinophilia was defined as a value of >0.4 × 10^9^ per liter measured a maximum of 3 months before or a maximum of 3 months after the assessment of *S. stercoralis* IgG.

### Laboratory diagnostics.

The serostatus test was performed in a reference laboratory by using an in-house-developed ELISA to determine an IgG1/IgG4 antibody response against a homogenate of L3 stage-larvae of *S. stercoralis*. An IgG titer of 1:40 or higher was considered positive, as stated by the reference laboratory. A sensitivity of at least 95% has been described using this cutoff value.^20^^,^^21^

## STATISTICAL ANALYSES

Descriptive analyses were performed to summarize demographic data and clinical characteristics. Continuous variables are presented as mean and SD or median and range. Categorical variables were summarized as absolute numbers or frequencies (percentages).

## RESULTS

### Demographics.

In total, 379 patients were included in this study. Of these 379 patients, 319 (84.2%) were immunocompromised, and 60 (15.8%) were screened because of the proposed initiation of immunosuppressants. The median age was 46 (interquartile range [IQR]: 32–58), and 222 patients were male (58.6%). The most frequent reason for immunosuppression was HIV infection (*n* = 150; 47.0%), followed by hematological malignancy (*n* = 71; 22.3%) and immunosuppressive medication (*n* = 61; 19.1%). One patient had two immunocompromising conditions. The country of origin was the Netherlands for 155 patients (40.9%), and two patients (0.5%) originated from other nonendemic countries. A total of 216 patients (57%) were from endemic countries, and for six patients (1.6%), the country of origin was unknown. The most common endemic countries were Suriname (*n* = 28), Indonesia (*n* = 18), the Dutch Caribbean (*n* = 11), and Thailand (*n* = 11).

### Screening for *S. stercoralis* IgG.

*Strongyloides stercoralis* IgG test results were positive in 17 of 379 (4.5%) patients. The median *S. stercoralis* IgG titer was 1:40 (IQR: 1:40–1:320), and the median eosinophilia count was 0.14 × 10^9^ per liter (IQR: 0.07–0.51). Eosinophilia was present in seven (41.1%) patients. All patients with positive serology results were born in an endemic country, and none of them developed strongyloides hyperinfection syndrome.

The most frequently reported reasons for screening were travel history (57.8%), endemic country of origin (57.3%), and (start)immunosuppressives (45.0%; multiple options possible).

The duration of travel was known for only 43/219 (21%) patients. Nine patients traveled for less than 4 weeks, eight patients traveled for 4–12 weeks, and 26 patients traveled for more than 3 months. The purpose of travel was known for 91/219 patients (42%) and was as follows: visiting relatives for 47 patients (52%) and tourism or work-related travel for 22 patients (24%).

For 29 patients (7.7%) screening was performed without any relevant country of birth or travel history. None of them had positive *S. stercoralis* IgG test results. The most frequent reasons for the presence of *S. stercoralis* IgG in this group without exposure included (start)immunosuppressives (*n* = 15), the presence of eosinophilia (*n* = 9), and the presence of symptoms (*n* = 6). When comparing patients with positive and negative serology results, the reasons for screening were different (Table 1).

### *Strongyloides* diagnostics over time.

**Figure 2.**
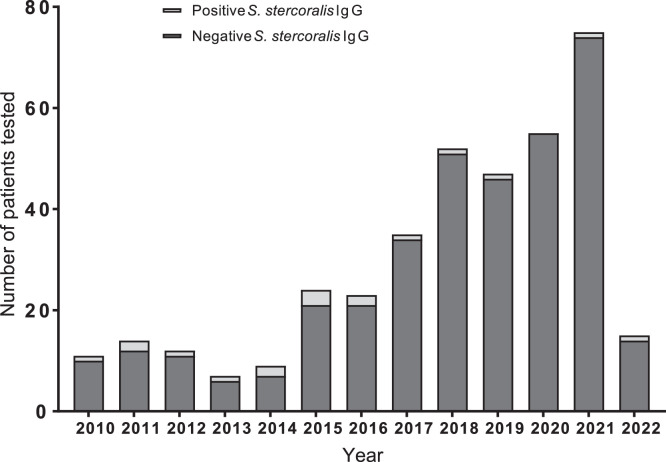
Number of patients tested from January 2010 to April 2022. The year 2022 is not representative because data are limited to only the first 4 months, during which one patient with a positive result was found.

**Figure 3.**
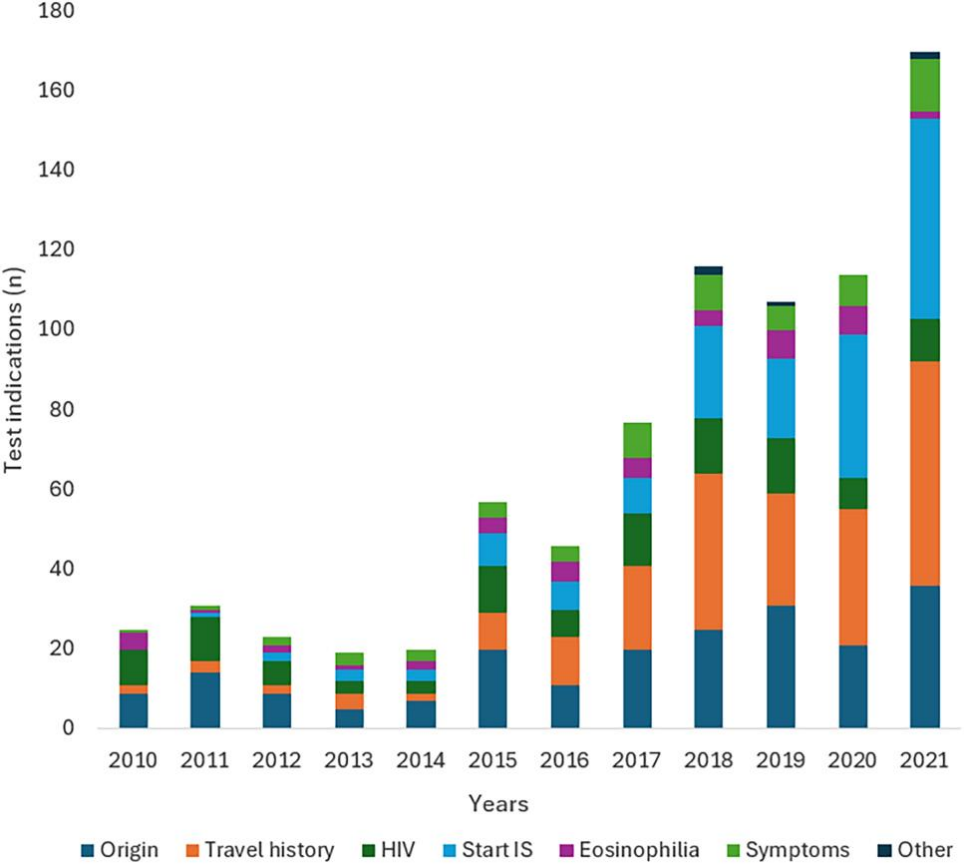
Indications for testing *S. stercoralis* IgG from January 2010 to December 2021. IS = immunosuppression.

Since 2014, the number of diagnostic tests for *S. stercoralis* IgG has increased, rising from nine diagnostic tests in 2014 to 75 tests in 2021 (Figure 2). Over time, more people have been screened because of travel history and (start)immunosuppression (Figure 3). However, the number of positive test results has remained approximately the same, with an average of one positive test result per year.

## DISCUSSION

Within our cohort of immunocompromised patients and those expected to be immunocompromised who were screened in a tertiary hospital in the Netherlands, *S. stercoralis* IgG testing generally yields a seroprevalence of 4.5%. Although this yield might be relatively low compared with the rates in high-endemic countries, it is a significant finding for several reasons.

Firstly, *S. stercorali*s is not endemic in the Netherlands, and the estimated prevalence of *S. stercoralis* is less than 0.1%.^19^ The prevalence in our cohort is substantially higher than that of the general population. This high prevalence highlights that screening is also relevant in certain patient groups in nonendemic countries. Secondly, infection with *S. stercoralis* could potentially lead to strongyloides hyperinfection syndrome, a condition associated with high mortality rates. Moreover, treating infection with *S. stercoralis* is easy and well-tolerated. Therefore, identifying and treating infection with *S. stercoralis* in immunocompromised patients is an effective preventive measure for reducing the risk of strongyloides hyperinfection syndrome.

Our data also suggest the importance of identifying the right immunocompromised patients for screening. In our cohort, all patients with positive *S. stercoralis* serology results had previously lived in endemic areas. We did not identify any patients with positive serology results who only visited endemic areas for vacation or who had not been in known endemic countries. Hence, we conclude that in our cohort, travelers to endemic areas are at a much lower risk for chronic strongyloidiasis than patients who originate from or have lived in endemic areas.

In patients who were born in or have lived in endemic countries, the seroprevalence of *S. stercoralis* IgG was 7.8%; the yield of screening would be almost doubled if we solely screened these patients. A previous study revealed the cost-effectiveness of screening migrants from endemic countries and supports the focus on screening patients from endemic countries.^22^

The prevalence of *S. stercoralis* IgG among travelers has been described in the literature but is low. One prospective study on the prevalence of a newly acquired *S. stercoralis* infection in short-term Dutch travelers (i.e., median travel duration of 21 days) revealed a prevalence of 0.25%. Factors associated with infection included previous travel to an endemic country, travel to Asia, and travel for work or to visit friends or relatives.^23^ Two other studies on long-term travelers also revealed low infection rates: 0.25% and 0.5%. Based on these data, the authors do not recommend screening asymptomatic healthy travelers.^24^^,^^25^

However, this recommendation does not apply to immunocompromised travelers who are at risk of developing strongyloides hyperinfection syndrome. Current guidelines for screening strongyloidiasis are ambiguous in their recommendations for screening immunocompromised patients. Some guidelines recommend screening patients from endemic areas, whereas others extend this recommendation to those who have traveled to endemic areas and those who have unexplained eosinophilia.^14^^,^^26^^,^^27^ Some guidelines recommend screening at-risk patients, whereas others recommend considering screening for these patients.^15^^,^^28^

Given the high seroprevalence in our cohort and the risk of strongyloides hyperinfection syndrome, we recommend screening immunocompromised patients. However, over the past 12 years, the number of tests ordered in our hospital has increased with decreased screening efficacy. The rise in ordered tests suggests that clinicians are more aware of strongyloidiasis but at the cost of screening efficacy. Hence, we must optimize our screening strategy. Most importantly, the threshold for screening travelers without additional risk factors could be improved.

Based on our data, we recommend screening for *S. stercoralis* in all immunocompromised patients from high-endemic countries (prevalence of ≥5% amongst the general population). For patients with a significant travel history, we recommend default screening for those who are about to undergo severe immunosuppression, such as hematopoietic stem cell or solid organ transplant recipients. For patients who are starting less severe immunosuppression, including those with chronic inflammatory diseases or malignancies, default screening is not advised. Instead, screening should be individualized according to our data and the literature based on travel characteristics, such as visiting friends or relatives (VFR) or direct contact with soil.^4^ Because two studies on long-term travelers have revealed low *S. stercoralis* infection rates (0.25% and 0.5%), we do not recommend using duration of stay as a criterion for deciding whether to screen.^24^^,^^25^

In immunosuppressed patients, physicians should be aware of the increased risk of false negative results associated with serological screening. A negative serological test result does not rule out *Strongyloides* infection, and clinicians should remain alert for this diagnosis if the patient develops relevant symptoms. Moreover, additional stool tests may be considered to improve diagnostic yield in case of a suspected infection.^19^^,^^29^

Patients who have never visited endemic areas should not be screened. Theoretically, screening these patients is not justified, and our data support this by revealing no positive results in such cases. To enhance appropriate screening practices, an understanding of the epidemiology of *S. stercoralis* is of vital importance.

### Strengths and limitations.

One of the main strengths of this study is that it is one of the few that has been conducted on a Western population, encompassing many patients over 12 years of *S. stercoralis* testing. Our data provide valuable insights into the yield and efficacy of our current screening strategy. However, our study has several limitations. Because of the retrospective study design, we only had access to data from patients screened during routine clinical care. As a result, we cannot provide seroprevalence data for all immunocompromised patients because we did not include every immunocompromised patient from our hospital. Additionally, the data are influenced by current screening protocols and practices.

Another limitation is that we used serological screening for Strongyloidiasis. In immunocompromised patients, this test can yield false negative results. Hence, we may have missed individuals with strongyloidiasis who could have been diagnosed by using another test (e.g., a Baermann or polymerase chain reaction [PCR] on feces). As a result, the observed seroprevalence may be underestimated. However, these possibly missed individuals concern only patients who were already immunocompromised when they were exposed to *S. stercoralis*. Patients exposed to *S. stercoralis* before becoming immunosuppressed could still mount a good immune response with detectable antibodies. Because a negative PCR result on feces cannot exclude an infection, we believe that serology remains a valuable tool for screening immunocompromised patients.

## CONCLUSION

In conclusion, screening for *S. stercoralis* IgG reveals a seroprevalence of 4.5% within our cohort of (pre)immunocompromised patients at a tertiary hospital in the Netherlands. Focusing screening on patients who have lived in endemic regions and reducing focus on screening travelers will improve diagnostic yield. Therefore, we recommend screening all immunocompromised patients who have lived in high-endemic countries. For those with a relevant travel history, we recommend default screening for highly immunocompromised patients. For less severely immunocompromised patients, a more individualized approach based on travel characteristics, such as VFR and direct contact with soil, is recommended.
